# Depression during pregnancy and preterm delivery: a prospective cohort study among women attending antenatal clinic at Pumwani Maternity Hospital

**DOI:** 10.1186/s12991-018-0202-6

**Published:** 2018-07-25

**Authors:** Kingi Mochache, Muthoni Mathai, Onesmus Gachuno, Ann Vander Stoep, Manasi Kumar

**Affiliations:** 1 Department of Psychiatry, University of Nairobi, Nairobi, Kenya; 2 Dept of Obstetrics and Gynecology, University of Nairobi, Nairobi, Kenya; 3 Psychiatry & Behavioral Sciences and Epidemiology, University of Washington, Nairobi, Kenya; 4 Research Dept of Clinical, Health and Educational Psychology, University College London, London, UK

**Keywords:** Antenatal depression, Prospective cohort, Preterm delivery, Low-income country

## Abstract

**Background:**

Preterm birth occurs among 9.6% of births worldwide and is the leading cause of long-term neurodevelopmental disabilities among children and also responsible for 28% of neonatal deaths. No single etiological factor is responsible for preterm birth, but various risk factors have been identified. Prior studies have reported that compromised maternal mental health occurring during pregnancy may lead to various adverse obstetric outcomes.

**Objective:**

To determine whether antenatal depression is significantly associated with preterm delivery in a low resource hospital sample from suburbs of Nairobi.

**Methods:**

292 women attending the antenatal clinic at Pumwani Maternity Hospital in Nairobi meeting the study criteria were recruited. The Edinburgh Postnatal Depression Scale was administered to screen for depression. A clinical cutoff score of 10 and above was regarded as possible depression. Thereafter, a clinical interview together with the Patient Health Questionnaire-9 was administered to evaluate the participants on DSM-V criteria for major depressive disorder. Only 255 of the women were successfully followed-up to delivery with an attrition rate of 12.7%. Records of gestation at delivery and birth weight were collected at second contact.

**Data analysis:**

Preterm birth was associated with various demographic, psychosocial and medical variables. Relative risks were estimated via log binomial regression analysis to determine whether depression was a risk factor for preterm birth.

**Results:**

Of the 255 participants, 98(38.4%) found to have depressive symptoms and 27(10.7%) delivered preterm. The risk of delivering preterm was 3.8 times higher among those with depressive symptoms.

**Conclusion:**

There is a positive association between antenatal depression and preterm delivery. This highlights the importance of screening for mental health challenges in the antenatal period as a means to reduce adverse obstetric outcomes.

## Background

Preterm birth is defined as a live birth delivered before 37 completed weeks of gestation [1]. It occurs among 9.6% of births worldwide with a higher rate of 12.5% amongst the low- and middle-income countries [2]. In Kenya, 12.3% of the live births in the year 2010 were preterm [1]. Preterm birth contributes to 28% of all neonatal mortalities and continues to be an economic healthcare burden, putting the surviving children at a seven times higher risk of various morbidities [3, 4]. Therefore, it is also the leading cause of long-term neurodevelopmental disabilities [5].

Adolescent pregnancies, being of African ethnicity, short birth spacing intervals and underweight pre-pregnancy weights have been shown to moderately predispose women to preterm labor [6]. Micronutrient deficiencies of both iron and folic acid also show weak evidence, whereas previous preterm birth, multiple gestation, in vitro fertilization, anatomic anomalies on the uterus, cervix and placenta, multiple miscarriages and abortions, fetal anomalies, physical trauma and injuries are strongly associated with preterm delivery [6, 7]. Mental health disturbances due to exposure to intimate partner violence (IPV) [8], or stressful life events [9], and perceived lack of social support have the potential to trigger preterm labor resulting in preterm birth. Pre-eclampsia, a condition where high blood pressure occurs in pregnancy accompanied by other symptoms potentially leads to early induction of labor or early cesarean section delivery thereby preterm birth [6].

Various hypotheses have been thought to link depression to preterm delivery confounded by an elevated risk of substance abuse (such as smoking, alcohol intake), poor nutrition and antenatal care attendance among those with depression; these factors being associated with preterm labor and may actually be mediators between depression and various adverse neonatal outcomes with a focus on preterm birth [9]. Additionally, patients with psychiatric disorders are more likely to engage in risky sexual behaviors that increase their risk of acquiring sexually transmitted infections which in turn increase their risk for preterm delivery [10, 6]. Women with good social support, high levels of education and higher socio-economic status, though suffering from depression, may be less likely to deliver preterm babies due to the moderating effects of diligent attendance at antenatal clinic, better maternal health awareness and taking good nutrition [6]. Physiologically, experiencing a heightened level of stress along with depression may in turn activate inflammatory pathways involving maternal cortisol that may also lead to premature delivery [11, 12]. In sub-Saharan Africa, depression during pregnancy varies widely ranging between a prevalence of 8.1% in Nigeria [13], 26.6% in Ghana [14] to 41% in South Africa [15] yet only less than 10% of all those with depression are diagnosed or managed in a lifetime [16]. There are currently no statistics published on the prevalence of depression during the antenatal period in Kenya yet. Research findings during the last decade on the links between antenatal depression and preterm births have been inconsistent. With some studies suggesting no association at all [17] and others reporting a strong positive association [18, 19]. The conclusions drawn may be influenced by various factors including geographical location (tropics versus temperate areas), the socioeconomic status of the population studied and the method of detection and classification of depression. There is no direct cause of preterm birth and there has been an effort to demystify various risk factors that lead to myriad of inflammatory processes associated with it [20].

The purpose of this pilot study was to explore associations between depression during pregnancy and preterm delivery focusing on a sample from a public hospital catering to vulnerable and resource-deprived women population in Nairobi, Kenya.

## Methods and materials

### Study population and design

This was a prospective cohort study carried out in Pumwani Maternity Hospital, the largest public maternity hospital in Nairobi that serves mainly a low-and low-middle income population. The researcher in person approached all consecutive women attending the antenatal clinic between August and October 2015 for possible participation in the study. The research design included the following exclusions:

- Being in their first and second trimester of pregnancy
- Those without telephone access for post delivery feedback
- Those unable to or opting not to give informed written consent.
- Women who had conceived in less than 6 months since prior delivery
- Those with multiple gestations.

The last two categories were excluded because they are already at higher risk of preterm delivery [6].

The sample size was calculated using *Epi Info version 7*. To assess the association of preterm birth with antenatal depression, we used an estimated prevalence of antenatal depression at 26.6% [21], prevalence of preterm birth of 12.3% [22] using a power of 80% and aiming to detect a risk ratio of 2.32 [23], we settled for an enrollment of 262 women.

### Ethical considerations

The study was approved by the Kenyatta National Hospital/University of Nairobi Ethical Research Committee (Approval No P107/02/2015) and authorized by the Medical Superintendent of Pumwani Maternity Hospital in July 2015.

### Study procedure

The researcher explained the study orally to the potential participants after which some gave informed consent. The eligibility criterion was then applied and those that fit were given a questionnaire that detailed sociodemographic data. Each participant was given a study number and asked for the information of two contacts (the participant’s own and next of kin’s telephone number). The participants’ past obstetric, medical and psychiatric history were collected including data pertaining to behavior, experience of stressful events, IPV and social support during the pregnancy. The researcher abstracted the hemoglobin levels from each participant’s antenatal profile book and recorded the pregnancy gestation from their ultrasound reports conducted in the third trimester. A physical examination was performed assessing the blood pressure and pregnancy fundal heights by the resident antenatal nurse attending to patients at the Pumwani maternity clinic as a research assistant. All the women filled out the 10-item self-administered Edinburgh Postnatal Depression Scale (EPDS), an internationally validated tool used to screen for perinatal depression [24, 25]. Both the English and the recently validated Kiswahili versions were used depending on the preference of the participant [26]. Though bearing the term postnatal, the tool takes into consideration the somatic symptoms that are common both in the perinatal period and are also indicative of depression, such as appetite changes, sleep pattern disruption and fatigue [27, 25]. Out of a maximum score of 30, mothers who score > 13 are categorized as having high depression scores [24]. We however employed a lower cutoff score of 10 to increase sensitivity and avoid missing any participants with probable depression [28]. All patients scoring ≥ 10 underwent a clinical interview by the researcher (then a postgraduate resident in psychiatry) to evaluate the women on the nine criteria symptoms of major depressive disorder on the DSM5 and thereby classified using the provider-initiated Patient Health Questionnaire (PHQ-9) that carries the same core symptoms [29]. Clarifications from participants was sought to differentiate the aforementioned somatic symptoms of depression from normal pregnancy-related symptoms. Women with a score above ≥ 5 were considered to have symptoms of depression and were therefore referred for specialized care.

Participants were contacted on telephone by the researcher around and after the estimated date of delivery to obtain the exact date, mode of delivery and the infant birth weight. Gestation at delivery was calculated using the best obstetric estimate that was extracted as an average of the fundal height measured during antenatal visits, last menstrual period date recorded on the antenatal profile book, birth weight and dates by ultrasound [2]. Preterm delivery was defined as birth before 37 completed weeks of gestation [22].

### Statistical analysis

The collected data were then analyzed using *STATA version 12.1*-*stata corporations*, Texas, USA. We presented descriptive for sample sociodemographic characteristics, prevalence of depression in preterm births and full-term births. Relative risks were estimated via log binomial regression analysis to determine the associations between preterm delivery with various sociodemographic, behavioral and medical characteristics.

## Results

**Fig. 1:**
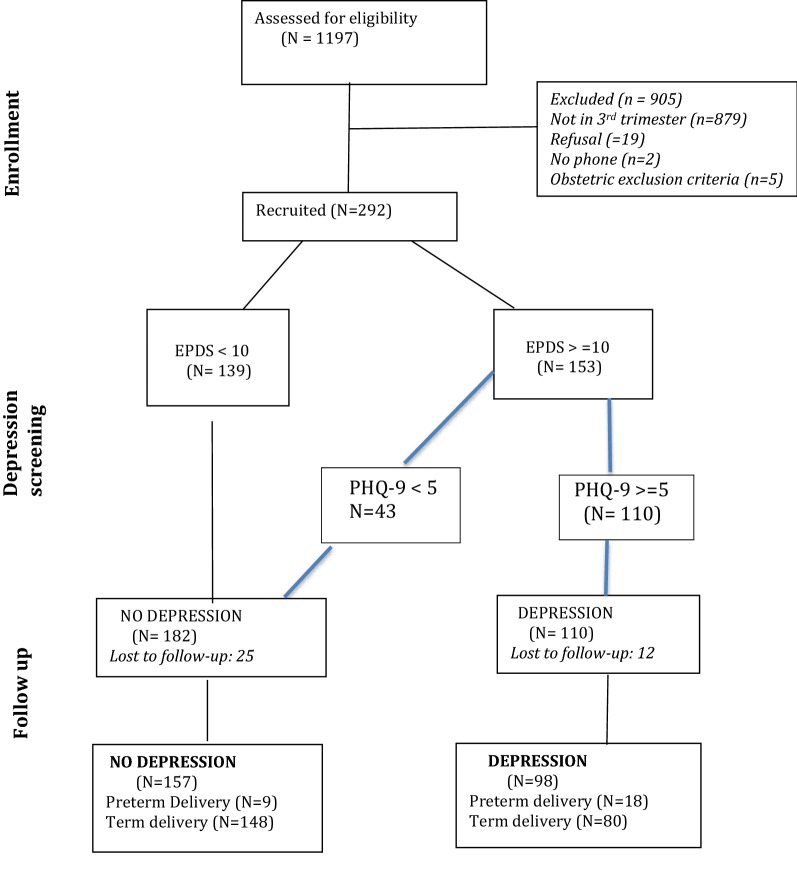
A diagram showing the flow of participants through each stage of the prospective cohort study on depression and preterm delivery at Pumwani Maternity Hospital

Between August 2015 and October 2015, all the women attending antenatal clinic at Pumwani maternity Hospital (*n* = 1197) were approached and screened for inclusion into the study; 905 did not meet the study criteria as 879 were in their first or second trimester, 19 preferred not to take part, two did not have reliable telephone access and were consequently not eligible, three were carrying multiple gestation and two had conceived in less than 6 months from the prior pregnancy, therefore excluded from participation. A total of 292 women were recruited into the study (see Fig. 1).

Edinburgh postnatal depression scale was used to screen for depression and a cutoff of 10 points employed and those scoring below 10 (*n* = 139) were considered to have no depression symptoms. Those that scored 10 and above (*n* = 153) underwent a clinical interview with objective scoring using a provider administered PHQ-9 depression scale scoring as follows: No depression (below 5 [*n* = 43]), mild depression (between 5 and 9 [*n* = 84]), moderate depression (10–19 [*n* = 26]) and severe depression (> 19 [*n* = 0]). A total of 182 had no depression and 110 had depression after both tools were employed. Out of these 12.7% (*n* = 37) were lost to follow-up and 87.3% (*n* = 255) were successfully followed and their data reported in the paper. In total, out of the 255 participants in the analysis 61.6% (*n* = 157) had no depression and 38.4% (*n* = 98) had depression. Those with depression were all referred to Mama Lucy hospital (a public Nairobi County Hospital) for further management of these symptoms.

### Socio-demographics of the study sample

In the study sample as shown in Table 1, 72.9% (*n* = 186) of our participants were women of ages 20–29 years, 83.1% (*n* = 212) were married and 75.7% (*n* = 193) had a secondary school education and above. About 89.0% (*n* = 227) of our participants earned less than Ksh. 35,000 (approx. 338 USD) with 10.9% (*n* = 28) earning less than Ksh. 5000 (USD 49 in 2015) a month. About 11.8% (*n* = 30) had experienced preterm births previously.

**Table 1:** Maternal demographics of patients screened for depressive symptoms

Demographic	Total no. *N* = 255	No depressive symptoms *n* = 157	Presence of depressive symptoms *n* = 98
Age (years)			
< 19	18	7(4.5)	11(11.2)
20–29	186	115(73.2)	71(72.5)
> 30	55	35(22.3)	16(16.3)
Marital status			
Married	212	133(84.7)	79(80.6)
Single	43	24(15.2)	19(19.3)
Education level			
No formal education	5	2(1.3)	3(3.1)
Primary education	57	32(20.4)	25(25.5)
Secondary education	142	87(55.4)	55(56.1)
Higher education	51	36(22.9)	15(15.3)
Monthly income			
Less than 50 USD	26	14(8.9)	12(12.2)
50–100 USD per month	60	33(21.0)	27(27.6)
100–350 USD per month	141	90(57.3)	51(52.0)
> 350 USD per month	28	20(12.8)	8(8.1)
Previous preterm delivery			
Yes	30	20(12.7)	10(10.2)
No	225	137(87.3)	88(89.8)
On antidepressant medication			
Yes	3	1(0.6)	2(2.0)
No	252	156(99.4)	96(98.0)
Week of first antenatal visit			
< 12 weeks (first trimester)	33	23(14.6)	10(10.2)
12–28 weeks (2nd trimester)	214	129(82.2)	85(86.7)
> 28 weeks (3rd trimester)	8	5(3.2)	3(3.1)

Our assessment found that 38.4% (*n* = 98) demonstrated depressive symptoms and three of these women were on antidepressant medication namely amitriptyline. Only one of the three on the medication reported feeling better with no current distress symptoms.

Overall, what worried us is the unusual practice of poor women to delay antenatal service access until much later. Our participants attended their first antenatal clinic during various trimesters with only 12.9% (*n* = 33) attending it in their first trimester.

### Preterm birth

Among the 27 participants (10.6%) who delivered preterm birth babies, one was under 19 years old, another participant had no formal education, one had experienced intimate partner violence and one smoked cigarettes regularly during pregnancy. None of these participants reported lack of social support. Two participants had anemia (Hb < 10 gm/dl) and two were taking alcohol during pregnancy. Among participants who earned less < 50 USD per month, 6 were found to have a 2.5 times higher risk of preterm birth. There were thirteen participants who had experienced a stressful life event during pregnancy and we found that the stress predisposed them to a 1.31 higher risk of preterm birth. Three participants had delivered preterm previously and they had no significant risk contributing to the current preterm birth (RR = 2.93). Five of our participants were found to have high systolic blood pressure (measuring > 140 mmHg) and we found that these women had a two times higher risk of preterm birth (RR = 2.09). The abovementioned findings are clinically relevant, however, these were not found to be statistically significant. We suspect that the small sample size has contributed to confidence intervals. The main finding of our study is the presence of depression among the participants who delivered preterm (*n* = 19) who were at a three times higher risk of delivering preterm births than those with no depressive symptoms (RR = 3.80, 95% CI 1.73–8.37) (Table 2).

**Table 2:** The association between various demographic, psychosocial and medical factors with preterm delivery

Factor	Preterm	Full Term	RR (95% CI)	*P* value	Adjusted RR	*P* value
	*N* = 27	*N* = 228				
Demographic factors						
A. Age (years)						
< 19	1(3.7)	17(7.5)	0.57(0.08–4.22)		2.34(0.32–17.05)	0.403
20–24	10(37.0)	93(40.8)	1.00(Ref)	0.864	1.00(Ref)	
25–29	9(33.3)	74(32.5)	1.12(0.48–2.62)		2.36(0.3–18.48)	0.412
30–34	6(22.2)	35(15.4)	1.51(0.58–3.89)		4.31(0.53–35.14)	0.172
35–40	1(3.7)	9(3.9)	1.03(0.15–7.27)		2.53(0.16–39.98)	0.511
B. Highest education						
Tertiary (university/college)	3(11.1)	48(21.1)	0.52(0.16–1.72)		0.40(0.1–1.67)	0.212
Secondary/high school	16(59.3)	126(55.3	1.09(0.47–2.51)	0.593	0.90(0.34–2.4)	0.839
Primary school	7(25.9)	50(21.9)	1.00(Ref)		1.00(Ref)	
No formal education	1(3.7)	4(1.8)	1.77(0.29–10.92)		1.43(0.36–5.62)	0.612
C. Monthly income (USD)						
< 50	6(22.2)	20(8.8)	2.50(1.04–6.00)		0.52(0.22–1,24)	0.142
50–99	5(18.5)	55(24.1)	0.90(0.34–2.43)		0.35(0.11–1.08)	0.068
100–349	13(48.1)	128(56.1	1.00(Ref)	0.259	1.00(Ref)	
> 350	3(11.1)	25(10.9)	1.16(0.35–3.81)		0.64(0.14–2.96)	0.572
D. Psychosocial factors						
1. Stressful life event						
No	14(51.9)	135(59.2	1.00(Ref)	0.463		
Yes	13(48.1)	93(40.8)	1.31(0.64–2.67)		1.18(0.54–2.59)	0.673
2. Perceived presence of social support						
Yes	27(100.0)	214(93.9	NA	0.185		
No	0(0.0)	14(6.1)	NA		NA	NA
3. Experience of IPV						
No	26(96.3)	216(94.7	1.00(Ref)	0.728		
Yes	1(3.7)	12(5.3)	0.72(0.10–4.89)		0.44(0.07–2.91)	0.392
4. Alcohol use in pregnancy						
No	25(92.6)	221(96.9	1.00(Ref)	0.248		
Yes	2(7.4)	7(3.1)	2.19(0.61–7.86)		1.95(0.63–6.02)	0.247
5. Cigarette smoking in pregnancy						
No	26(96.3)	224(98.2	1.00(Ref)	0.49		
Yes	1(3.7)	4(1.8)	1.92(0.32–11.56)		1.25(0.23–6.7)	0.795
E. Medical factors						
1. Previous preterm						
No	24(88.9)	201(88.2	1.00(Ref)	0.911		
Yes	3(11.1)	27(11.8)	0.94(0.30–2.93)		0.87(0.26–2.95)	0.829
2. Hemoglobin level (mmol/l)						
> 10	25(92.6)	210(92.1	1.00(Ref)	0.929		
< 10	2(7.4)	18(7.9)	0.94(0.24–3.69)		0.80(0.15–4.29)	0.795
3. Systolic blood pressure						
Below 140 mmHg	22(81.5)	208(91.2	1.00(Ref)	0.107		
Above/equal to 140 mmHg	5(18.5)	20(8.8)	2.09(0.87–5.04)		2.20(0.93–5.19)	0.072
F. Depressive symptoms						
No depressive symptoms	8(29.6)	149(65.4	1.00(Ref)	< *0.001*		
Present depressive symptoms	19(70.4)	79(34.6)	3.80(1.73–8.37)		3.56(1.7–7.48)	*0.001*

Significant *P* values are in italics

## Discussion

Among the 255 participants who participated in our study, 38.4% (*n* = 98) had symptoms of depression. This figure closely matches to that found in a study by Rochat et al. in South Africa where 41.1% of the antenatal women reported depression and an another study by Bindt et al. found a depression prevalence of 26.6% in Ghana, which is a country similar to Kenya in terms of its geography, economic status and population characteristics [15, 21]. This difference between the studies may be attributed to the fact that our study participants were all from a lower socioeconomic status, living in a peri-urban environment and the study took place during a time when Pumwani Maternity Hospital was undergoing a staff go-slow period. Consequently, only desperate mothers attended the antenatal clinic in this free government facility. This was already clinically speaking a group at higher risk for depression as financial difficulty predisposes to poor mental health [16].

A large majority of our participants (72.9%) were 20–29 years old, 83.1% were married and 75.7% of women had an education above secondary school, despite this about 89% earned less < 350 USD per month translating to less than a dollar a day, a figure considered emblematic of the poverty line as defined by the World Bank [30].

Only two of the participants with symptoms of depression were on medication or aware of their illness, leaving the 96 participants with depressive symptoms with little to no information or access to treatment. This concurs with the WHO factsheet that less than 10% of those with depression in sub-Saharan Africa are diagnosed or managed in healthcare in their lifetime [16].

In an ideal situation, every pregnant woman should attend antenatal clinic the moment they discover that conception has taken place and ideally this visit should take place within the first trimester of pregnancy to detect and deal with any potential obstetric complications, but this has been a hurdle in Kenya with previous studies reporting barely 15% attending antenatal clinic in the first trimester of pregnancy mirrored in this study by 12.9% (*n* = 33) who had had attended clinic before 12 weeks of gestation [31].

The participants who delivered preterm in our sample were 10.6% (*n* = 27) which is a slightly lower rate that the national prevalence of 12.3% [22]. We attribute this lower rate to our exclusion criteria that exempted participants with known risk factors for preterm birth including the three who were carrying multiple gestation and two who had conceived in less than 6 months from prior delivery [32].

Various risk factors including being of a younger age (< 19 years), exposure to IPV, nicotine addiction, lack of social support, micronutrient deficiency (Hemoglobin level < 10 gm/dl), previous preterm birth and alcohol use during pregnancy did not yield a statistically significant association with preterm birth. Low socioeconomic status (< 50 USD per month) and high systolic blood pressure increased the risk of preterm birth twofold at RR = 2.50 and 2.90, respectively, and the experience of a stressful life event by RR = 1.31. These findings were not statistically significant (*P* > 0.05) as the sample size was too small, as these were calculated taking the prevalence of depression into account. The participants with symptoms of depression had a three times higher risk of preterm birth than those with no depressive symptoms (RR = 3.80, 95% CI 1.73–8.37). These findings are similar to a study by Woebong carried out in Ghana in 2014 using the PHQ-9 to assess for depressive symptoms that revealed a 1.32 times higher risk [14]. However, another study from Ghana by Bindt et al. conducted in 2013 found no association between depression and preterm birth [17].

Preconception weight, which is a risk factor for preterm delivery, could not be objectively ascertained as visits to preconception clinic is not the norm in Kenya.

Our study was not without its limitations. It was carried out in a hospital located in an urban low socioeconomic area in Kenya and thus may not be representative of all pregnant Kenyan women. The poor socioeconomic condition of our participants and absence of prenatal information makes it hard to attribute depression as the key risk factor for preterm birth in our study participants.

Hospital based sampling frame may be biased too as many severely depressed women may not visit antenatal clinic at all throughout their pregnancy and this mentally ill sub-group may not be fully captured in the study. Further studies need to control for these limitations and biases for a more robust estimate of how depression triggers preterm birth.

## Conclusions

Our study has found an association between depression and preterm delivery. Preterm birth is an adverse obstetric outcome in Kenya that has been the national focus of in efforts to reduce neonatal mortality rate (Kenya Vision 2030, Ministry of Health). Women who are depressed during pregnancy have a 3.8 times higher risk of delivering preterm than those who are not depressed. Addressing the mental health needs of women as they attend antenatal clinic may aid in reducing risks associated with preterm delivery. All women attending antenatal care should be screened for depression using a basic tool like the self-administered EPDS routinely. We wish that there would be newer findings from the follow-up study that could potentially do a follow-up on women from the preconception stage. This would mean taking records of preconception weights (BMI), include all socioeconomic classes of women and a first trimester ultrasound and follow-up these women into the post-partum period. Despite the time and costs associated with such an intensive work. we recommend an inquiry that extends into the perinatal context. This would eliminate all the study limitations cited above and provide more decisive conclusions. In our work, despite the gaps, we have been able to detect a strong impact of maternal depression on preterm delivery. We also hope awareness about mental health particularly depression and its management is generated in community health centers and amongst health workers so pregnant women can benefit from greater social engagement and mental health promotion.

## Abbreviations

ANC: antenatal clinic
BMI: body mass index
DSM-V: diagnostic and statistical manual 5th edition
EDD: estimated date of delivery
EPDS: Edinburgh Postnatal Depression Scale
IPV: intimate partner violence
LMIC: lower and middle income country
LMP: last menstrual period
MOH: Ministry of Health
PHQ-9: Patient Health Questionnaire 9
USD: United States Dollar
WHO: World Health Organization
