# Correction to: Depression during pregnancy and preterm delivery: a prospective cohort study among women attending antenatal clinic at Pumwani Maternity Hospital

**DOI:** 10.1186/s12991-018-0210-6

**Published:** 2018-10-05

**Authors:** Kingi Mochache, Muthoni Mathai, Onesmus Gachuno, Ann Vander Stoep, Manasi Kumar

**Affiliations:** 1 Department of Psychiatry, University of Nairobi, Nairobi, Kenya; 2 Dept of Obstetrics and Gynecology, University of Nairobi, Nairobi, Kenya; 3 Psychiatry & Behavioral Sciences and Epidemiology, University of Washington, Washington, USA; 4 Research Dept of Clinical, Health and Educational Psychology, University College London, London, UK

## Correction to: Ann Gen Psychiatry (2018) 17:31 10.1186/s12991-018-0202-6

Following the publication of the original article [1], the authors reported the following typesetting errors:

In the Background section, first paragraph, the last sentence should read, “It is also the leading cause of long-term neurodevelopmental disabilities [5]”.

In the Background section, third paragraph, the first sentence should read, “Various hypotheses have been thought to link depression to preterm delivery. An elevated risk of substance abuse (such as smoking, alcohol intake) poor nutrition and inconsistent antenatal care attendance among those with depression are factors associated with preterm labor and may actually be mediators between depression and various adverse neonatal outcomes with a focus on preterm birth [9].”

In the sub-section “Socio-demographics of the study sample”, the last paragraph should read, “Our participants attended their first antenatal clinic during various trimesters with only 12.9% (*n* = 33) attending it in their first as is recommended in obstetric practice.”

In the sub-section “Preterm birth”, the paragraph should read:

Among the 27 participants (10.6%) who delivered preterm birth babies, one was under 19 years old, another participant had no formal education, one had experienced intimate partner violence and one smoked cigarettes regularly during pregnancy. None of these participants reported lack of social support. Two participants had anemia (Hb < 10 gm/dl) and two were taking alcohol during pregnancy. Among participants who earned less < 50 USD per month 6 were found to have a 2.5 times higher risk of preterm birth. There were thirteen participants who had experienced a stressful life event during pregnancy and we found that the stress predisposed them to a 1.31 higher risk of preterm birth. Three participants had delivered preterm previously and they had no significant risk contributing to the current preterm birth (RR = 2.93). Five of our participants were found to have high systolic blood pressure (measuring > 140 mmHg) and we found that these women had a two times higher risk of preterm birth (RR = 2.09). The above mentioned finding though clinically relevant were not found to be statistically significant. We suspect that the small sample size has contributed to confidence intervals. The main finding of our study is the presence of depression among the participants who delivered preterm (n = 19) who were at a three times higher risk of delivering preterm births than those with no depressive symptoms (RR = 3.80, 95% CI 1.73–8.37).

In Table 1, the last column header should read, “Presence of depressive symptoms *n* = 98”.

In Table 2, the *P* value “< *0.001 and 0.001*” in the last two rows should be italics.

In the Discussion section, paragraph seven should read, “Preconception weight, which is a risk factor for preterm delivery, could not be objectively ascertained as visits to preconception clinic is not the norm in Kenya.”

In the Discussion section, the last sentence in paragraph eighth should read, “The poor socioeconomic condition of our participants and absence of prenatal information makes it hard to attribute depression as the key risk factor for preterm birth in our study participants.”

The last paragraph of the Discussion section should read, “Hospital based sampling frame may be biased too as many severely depressed women may not visit antenatal clinic at all throughout their pregnancy and this mentally ill sub-group may not be fully captured in the study. Further studies need to control for these limitations and biases for a more robust estimate of how depression triggers preterm birth.”

In the Conclusion section, the first sentence should read, “Our study has found an association between depression and preterm delivery. Preterm birth is an adverse obstetric outcome in Kenya that has been then national focus in efforts to reduce neonatal mortality rate (Kenya Vision 2030, Ministry of Health).”

In the Conclusion section, the third sentence should read, “Addressing the mental health needs of women as they attend antenatal clinic may aid in reducing risks associated with preterm delivery” and the last sentence should be, “We also hope awareness about mental health particularly depression and its management is generated in community health centers and amongst health workers so pregnant women can benefit from greater social engagement and mental health promotion.”

The original article [1] has been updated.
